# Nicotinamide Supplementation during the In Vitro Maturation of Oocytes Improves the Developmental Competence of Preimplantation Embryos: Potential Link to SIRT1/AKT Signaling

**DOI:** 10.3390/cells9061550

**Published:** 2020-06-25

**Authors:** Marwa El Sheikh, Ahmed Atef Mesalam, Muhammad Idrees, Tabinda Sidrat, Ayman Mesalam, Kyeong-Lim Lee, Il-Keun Kong

**Affiliations:** 1 Division of Applied Life Science (BK21 Plus), Gyeongsang National University, Jinju 52828, Korea; marwa.elsheikh@gnu.ac.kr (M.E.S.); ahmedatefmesalam@hotmail.com (A.A.M.); idrees1600@gnu.ac.kr (M.I.); tabindasidrat06@gmail.com (T.S.); 0920-0728@hanmail.net (K.-L.L.); 2 Department of Microbial Biotechnology, Genetic Engineering and Biotechnology Division, National Research Centre, Dokki, Cairo 12622, Egypt; 3 Department of Therapeutic Chemistry, Division of Pharmaceutical and Drug Industries Research, National Research Centre, Dokki, Cairo 12622, Egypt; 4 Department of Theriogenology, Faculty of Veterinary Medicine, Zagazig University, Zagazig 44519, Egypt; aymanmesalam@gmail.com; 5 King Kong Ltd., Gyeongsang National University, Jinju 52828, Korea; 6 Institute of Agriculture and Life Science, Gyeongsang National University, Jinju 52828, Korea

**Keywords:** Nicotinamide, AKT, SIRT1, hatching, maturation, ICM, autophagy, bovine blastocyst

## Abstract

Nicotinamide (NAM), the amide form of vitamin B3, plays pivotal roles in regulating various cellular processes including energy production and maintenance of genomic stability. The current study aimed at deciphering the effect of NAM, when administered during in vitro maturation (IVM), on the developmental competence of bovine preimplantation embryos. Our results showed that low NAM concentrations reduced the oxidative stress and improved mitochondrial profile, total cleavage and 8–16 cell stage embryo development whereas the opposite profile was observed upon exposure to high NAM concentrations (10 mM onward). Remarkably, the hatching rates of day-7 and day-8 blastocysts were significantly improved under 0.1 mM NAM treatment. Using RT-qPCR and immunofluorescence, the autophagy-related (Beclin-1 (BECN1), LC3B, and ATG5) and the apoptotic (Caspases; CASP3 and 9) markers were upregulated in oocytes exposed to high NAM concentration (40 mM), whereas only CASP3 was affected, downregulated, following 0.1 mM treatment. Additionally, the number of cells per blastocyst and the levels of SIRT1, PI3K, AKT, and mTOR were higher, while the inner cell mass-specific transcription factors GATA6, SOX2, and OCT4 were more abundant, in day-8 embryos of NAM-treated group. Taken together, to our knowledge, this is the first study reporting that administration of low NAM concentrations during IVM can ameliorate the developmental competence of embryos through the potential regulation of oxidative stress, apoptosis, and SIRT1/AKT signaling.

## 1. Introduction

Nicotinamide (NAM), also known as niacinamide, is engaged in therapeutics of various diseases including diabetes, tumor metastasis, Alzheimer’s, and multiple sclerosis [1]. It also regulates the survival of cells, as well as the control of microbial infections and inflammations [1]. In the field of assisted reproductive technology (ART), accumulation of reactive oxygen species (ROS) during the in vitro maturation (IVM) and culturing processes represents a significant barrier for efficient embryo development. This is mainly attributed to the major difference in the levels of oxygen and antioxidants between the in vivo environment and in vitro culturing conditions [2]. During the last decade, several biomolecules and bioactive compounds were verified as potent inducers for embryo development through reducing the pressure of ROS [2,3,4]. It was reported that NAM exhibits an antioxidant activity in addition to its role in the improvement of mitochondrial profile in different cells [5,6]; however, its actual role during the developmental process of preimplantation embryos is not completely clarified. Although, detrimental effects of high concentrations of NAM were observed in some studies [7,8,9], non-effective or even beneficial roles were also reported in other studies [10,11].

Autophagy is a metabolic process developed by cells for controlling apoptosis and removing the damaged organelles [12]. During oocyte maturation, the negative effect of hyper-activated autophagy was observed [13,14]. In addition, diminished autophagy following supplementation of imperatorin, a major bioactive furanocoumarin, to the in vitro culture medium, decreased apoptosis and enhanced the developmental competence of embryos [15]. On the contrary, the importance of autophagy for embryo development was also reported in other studies that used rapamycin, an inhibitor of the mammalian target of rapamycin (mTOR) [16,17].

Sirtuin-1 (SIRT1) is a nicotinamide adenine dinucleotide (NAD)-dependent deacetylase involved in protection of oocytes against aging-related oxidative stress and improving the quality/development of oocytes and primordial follicles through controlling the redox state [18,19,20]. Moreover, it regulates autophagy, mitochondrial function, and DNA repair, as well as the anti-inflammatory and anti-apoptosis processes [21]. SIRT1 exhibits its cellular activities via the transcription of various enzymes and signaling pathways including serine/threonine-specific protein kinase B (AKT) and mTOR [19]. On the other hand, the phosphatidylinositol 3-kinase (PI3K) and its downstream effectors AKT and mTOR regulate various cellular processes including apoptosis, development, proliferation, survival, and maintenance of pluripotency of embryos and stem cells [22,23,24,25,26]. PI3K mediates the activation of AKT at the Thr308 phosphorylation site via recruitment of phosphoinositide dependent kinase 1 (PDK1), whereas PDK1 can indirectly activate AKT at the Ser473 phosphorylation site by activating the rictor–mTOR complex 2 [27]. During oocyte maturation and embryo development, the role of PI3K/AKT/mTOR signaling and its modulation by SIRT1 were previously investigated [19,22,23,24].

At late stages of preimplantation embryo development, blastocysts comprising the two distinct parts, the outer trophectoderm (TE) and the inner cell mass (ICM), tend to hatch out of the zona pellucida. This precisely regulated process, the hatching, is crucial for survival, normal development, and implantation of embryos [28,29]. The critical roles of SIRT1 and AKT signaling in controlling the hatching process were reported [3,22,30]. Although the crosstalk between NAM and SIRT1/AKT was elucidated in different cancer cells [31,32], the potential linkage in the context of preimplantation embryo development is not yet reported. On the other hand, understanding the mechanisms behind the differentiation of zygotes into embryos containing all cell lineages will help to improve the quality of in vitro produced embryos, a critical step in ART. Generally, two differentiation processes frequently take place before the implantation of embryos. The first process is the development of blastocysts (TE and ICM), whereas the second is the segregation of ICM into primitive ectoderm (epiblast; Epi) and primitive endoderm (PE) that successively develop into the embryo and the extra embryo yolk sac, respectively [33]. The lineage’s specification fate of the ICM into Epi and PE is controlled by several transcription factors including the octamer-binding transcription factor 4 (OCT4), SRY-box transcription factor 2 (SOX2), GATA-binding protein 4 (GATA4) and GATA6 [34,35]. In addition, the impact of AKT/mTOR on regulating pluripotency was also addressed [26,30].

In the current study, the effects of NAM supplementation, during the IVM, on the mitochondrial profile and developmental competence of bovine embryos were investigated. The levels of ROS, apoptosis, autophagy, and pluripotency maintenance-related markers, as well as SIRT1/AKT signaling, were inspected at both transcriptional and translational levels.

## 2. Materials and Methods

### 2.1. Ethical Approval and Experimental Design

All experiments were performed under the regulation of the Institutional Animal Care and Use Committee, and according to the guidelines of Gyeongsang National University (Approval ID: GAR-110502-X0017). The current study underwent three main experimental set-ups. Experiment 1 was performed to study the effect of low NAM concentrations on the developmental competence of embryos. In this experiment, three different concentrations (0.01, 0.1, and 1.0 mM) of NAM in addition to an untreated control were used during the IVM process. After 20–22 h of treatment, cumulus cell expansion, oocyte maturation, ROS levels, day-4 total cleavage, 8–16 cell-stage embryos, day-7/day-8 blastocyst development, and hatching rates were assessed. Similarly, experiment 2 was executed using high concentrations (10, 20, and 40 mM) of NAM, and the same parameters of the first experiment were investigated. Based on the results of the first two experiments, experiment 3 was carried out using three groups corresponding to an untreated control, as well as high (40 mM) and low (0.1 mM) NAM exposure settings. In vitro matured oocytes and day-8 blastocysts were collected and used for mitochondrial profiling, immunofluorescence, and RT-qPCR analyses. All reagents were purchased from Sigma-Aldrich (St. Louis, MO, USA) unless otherwise specified.

### 2.2. Oocyte Collection and In Vitro Maturation

The ovaries of Korean native cows (Hanwoo) were collected at a local abattoir and transported to the laboratory in thermal bottles within 2 h of slaughter. Cumulus–oocyte complexes (COCs) were aspirated from the 2–8-mm-diameter follicles using 18-gauge needles and collected in 50-mL tubes containing TL-HEPES (10 mM of 4-(2-hydroxyethyl)-1-piperazineethanesulfonic acid (HEPES), 0.34 mM sodium biphosphate, 2 mM sodium bicarbonate, 114 mM sodium chloride, 10 mM sodium lactate, 3.2 mM potassium chloride, 0.5 mM magnesium chloride, 2.0 mM calcium chloride, 1 μL/mL phenol red, 0.1 mg/mL streptomycin, and 100 IU/mL penicillin). The COCs with at least three layers of cumulus cells were picked up under stereomicroscope (Olympus SZ51, Tokyo, Japan) and cultured in four-well plates containing 700 μL of IVM medium (TCM-199 supplemented with 1 μg/mL estradiol-17β, 10 μg/mL follicle stimulating hormone (FSH), 10 ng/mL epidermal growth factor (EGF), 0.2 mM sodium pyruvate, 0.6 mM cysteine, 0.1 mg/mL streptomycin, and 100 IU/mL penicillin), in the presence and absence of different NAM concentrations, at a density of around 50 oocytes per well for 20–22 h under 5% CO_2_ and 38.5 °C.

### 2.3. In Vitro Fertilization (IVF)

Fertilization of in vitro matured oocytes was carried out as previously described [23]. Briefly, frozen semen from a Hanwoo bull was thawed at 37 °C, then diluted in pre-warmed Dulbecco’s phosphate-buffered saline (DPBS), and centrifuged at 750× *g* for 5 min at room temperature. For sperm capacitation, the pellets were re-suspended in 500 μL of pre-warmed heparin (20 μg/mL) prepared in IVF medium (Tyrode’s lactate solution supplemented with 6 mg/mL bovine serum albumin (BSA), 22 mg/mL sodium pyruvate, 0.1 mg/mL streptomycin, and 100 IU/mL penicillin) and incubated at 38.5 °C and 5% CO_2_ for 15 min. Concentrated sperm was diluted in IVF medium to a final density of 1–2 × 10^6^ spermatozoa/mL, then 700 μL was added to COCs followed by incubation at 38.5 °C and 5% CO_2_ for 18–20 h.

### 2.4. In Vitro Culture and Development of Embryos

Following fertilization, cumulus cells were detached by successive pipetting, and the presumed zygotes were cultured in four-well plates containing 700 μL of complete synthetic oviductal fluid (SOF) medium [36] and incubated at 38.5 °C under 5% CO_2_. The cleavage rate and the number of 8–16 cell-stage embryos were recorded at day 4 post-fertilization (the day of fertilization was considered as day 0) before replenishing the medium and incubation for another four days. Blastocyst development and hatching rates were calculated at day 7 and day 8 post-fertilization. Day-8 blastocysts were either fixed in 4% paraformaldehyde and stored at 4 °C for use in staining experiments or kept at −80 °C for use in RNA extraction.

### 2.5. Assessment of Cumulus Expansion and Oocyte Maturation

To evaluate the process of cumulus expansion, around 50 COCs per group were morphologically tested under epifluorescence microscope (Olympus IX71, Tokyo, Japan), and the area of cumulus cell expansion (mm^2^) was calculated using ImageJ software (National Institutes of Health, Bethesda, MD, USA; https://imagej.nih.gov/ij/) by recording the surface area before and after the process of maturation. For oocyte maturation assessment, the COCs (around 30 per group) collected after 18–20 h from the onset of maturation were denuded by gentle vortex in 0.1% hyaluronidase and the first polar body extrusions were directly inspected under stereomicroscope. For confirmation, oocytes were permeabilized using 0.5% Triton X-100 for 20 min and stained with 4′,6-diamidino-2-phenylindole (DAPI). Oocytes were visualized under confocal laser scanning microscope (Olympus Fluoview FV1000, Tokyo, Japan). According to the morphology of the nuclear material, oocytes were classified as germinal vesicle stage (GV; immature) or metaphase II (MII; mature).

### 2.6. Estimation of Intracellular ROS Levels, Mitochondrial Content, and Distribution Pattern

Following maturation, oocytes (around 20 per group) were denuded of cumulus cells and incubated with 5 μM of the ROS indicator 2,7-dichlorodihydrofluorescein diacetate (H_2_DCFDA) for 15 min at 38.5 °C. After washing three times in PBS, oocytes were directly imaged using an epifluorescence microscope under 490-nm excitation and 525-nm emission wavelengths, and the fluorescence intensities were estimated using ImageJ. On the other hand, the mitochondrial content was assessed using Mito Tracker Green FM kit (Invitrogen, Carlsbad, CA, USA). Briefly, oocytes (around 20 per group) were washed in PBS and incubated with 125 nM Mito Tracker Green for 30 min at 38.5 °C. Oocytes were washed in PBS and examined under epifluorescence microscope while the fluorescence intensities were estimated using ImageJ and normalized to the control. For the mitochondrial distribution pattern, Mito Tracker deep Red FM (Molecular Probes, Eugene, OR, USA) was used as follows: oocytes (around 30 per group) were incubated with 100 nM Mito Tracker Red stain for 40 min at 38.5 °C, then washed in PBS, fixed in 4% paraformaldehyde for 15 min, and examined under epifluorescence microscope. The distribution pattern of mitochondria was classified either as aberrant where mitochondria were distributed peripherally/semi-peripherally or homogeneous where the mitochondria were dispersed throughout the cytoplasm.

### 2.7. Terminal Deoxynucleotidyl Transferase dUTP Nick-End Labeling (TUNEL) Assay

The TUNEL assay was performed using the In Situ Cell Death Detection kit (Roche Diagnostics, Indianapolis, IN, USA) according to the manufacturer’s instructions. In brief, paraformaldehyde-fixed blastocysts (around 20 per group) were washed three times in 0.3% polyvinylpyrrolidone (PVP) prepared in PBS (PBS-PVP) and permeabilized with 0.5% Triton X-100 and 0.1% sodium citrate for 30 min. Blastocysts were washed thrice and incubated with fluorescent-conjugated terminal deoxynucleotide transferase dUTP for 1 h at 38.5 °C. After washing, blastocysts were incubated with DAPI (1 µg/mL) for 15 min and examined under epifluorescence microscope where the TUNEL-positive cells, an indicator for DNA fragmentation, appeared as bright red spots.

### 2.8. RNA Extraction, Complementary DNA (cDNA) Synthesis, and Quantitative Reverse Transcription PCR (RT-qPCR)

Total RNA was extracted from matured oocytes (*n* = 50) and day-8 blastocysts (*n* = 5) using Arcturus PicoPure RNA Isolation Kit according to the manufacturer’s guidelines (Arcturus, Foster, CA, USA). After estimating the concentration and the purity of RNA using NanoDrop 2000c spectrophotometer (Thermo Fisher Scientific, Waltham, MA, USA), fixed concentrations of RNA (100 and 150 ng for blastocysts and oocytes, respectively) were subjected to cDNA synthesis using the iScript cDNA synthesis kit (Bio-Rad Laboratories, Hercules, CA, USA) as follows: 4 μL of 5× iScript reaction mixture, 1 μL of iScript reverse transcriptase, and 15 μL of RNA were mixed and incubated at 25 °C for 5 min, 42 °C for 30 min, and 85 °C for 5 min. The cDNA samples were diluted in nuclease-free water and kept at −20 °C until use. The RT-qPCR was carried out using iQ-SYBR Green Supermix (Bio-Rad Laboratories). In brief, 2 μL of forward and reverse primer mix was added to 5 μL of SYBR Green mix before adding 3 μL of cDNA. The mixture was exposed to qPCR reaction under the following conditions: initial denaturation at 95 °C for 3 min followed by 44 cycles of 95 °C for 15 s, 58 °C for 20 s, and 72 °C for 30 s. The genes of the study include: Caspase-3 (*CASP3*) and Caspase-9 (*CASP9*), B-cell lymphoma 2 (*BCL2*), autophagy-related genes (*ATG5*, *ATG7* and Beclin-1 (*BECN1*)), Microtubule-associated proteins 1A/1B light chain 3 beta (*MAP1LC3B*; *LC3B*), *SIRT1*, *PI3K*, *AKT*, *mTOR*, *GATA6*, *SOX2*, *OCT4*, glyceraldehyde-3-phosphate dehydrogenase (*GAPDH*) and β-actin (*ACTB*). The sequences of the used primers are listed in Table 1. Each cDNA sample was applied in duplicate, and the expression of different genes was calculated via the ΔΔCt method using *GAPDH* and *ACTB* as reference genes.

### 2.9. Immunofluorescence Analysis

The in vitro matured oocytes (around 30 per group) and day-8 blastocysts (around 20 per group), previously fixed in paraformaldehyde, were washed thrice in DPBS and permeabilized using 0.5% Triton X-100 for 20 min at room temperature. Samples were blocked in 10% fetal bovine serum and 3% BSA for 2 h then overnight incubated with the primary antibodies (Table S1, Supplementary Materials) at 4 °C. Samples were washed three times in PBS and incubated with fluorescein isothiocyanate (FITC)-, tetramethylrhodamine isothiocyanate (TRITC)-, Alexa Fluor 568-, and Alexa Fluor 488-conjugated secondary antibodies (Table S1, Supplementary Materials) for 90 min. Nuclei were stained with DAPI (1 µg/mL) for 15 min, followed by washing. Oocytes/embryos were investigated under confocal laser scanning microscope, whereas the optical densities were estimated using ImageJ and normalized to the control.

### 2.10. Statistical Analysis

Data were analyzed using GraphPad Prism version 6 (San Diego, CA, USA). The differences between groups were analyzed using either Student’s *t*-test or one-way ANOVA followed by Tukey’s multiple comparison. Data are presented as the mean values ± standard error of the mean (SEM), whereas *p*-values below 0.05 were considered statistically significant.

## 3. Results

### 3.1. Effect of NAM Supplementation during the IVM on Cumulus Expansion, Oocyte Maturation, and ROS Levels

**Figure 1:**
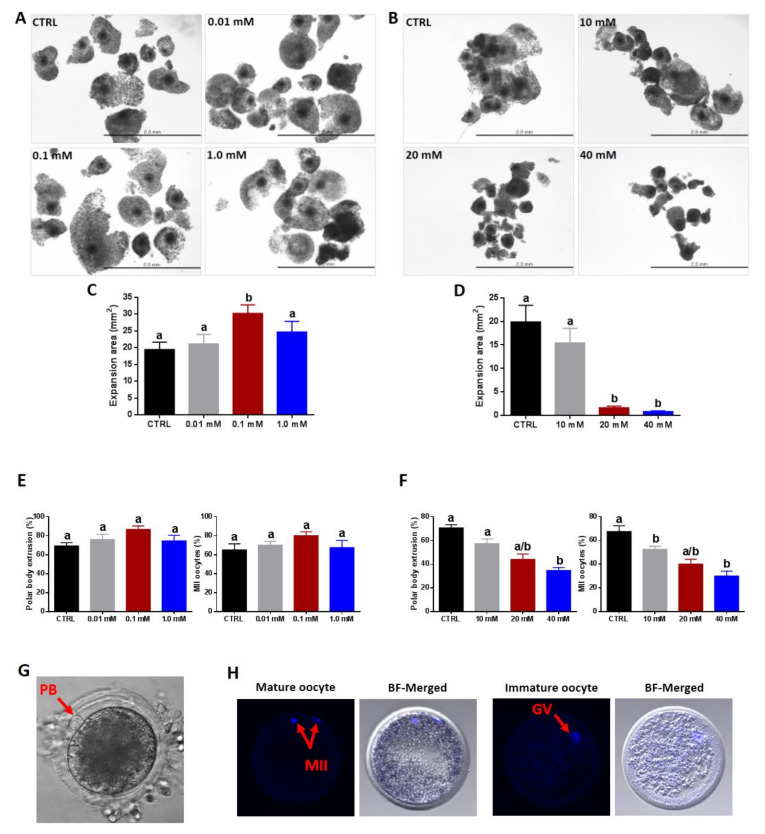
The effect of nicotinamide (NAM) on cumulus expansion and oocyte maturation. Light microscopy for cumulus–oocyte complexes (COCs) post-maturation in the presence of low (**A**) and high (**B**) NAM concentrations. The increase in the expansion area of COCs matured under low (**C**) and high (**D**) NAM concentrations. (**E**,**F**) Percentages of in vitro matured oocytes following NAM treatment. Representative images for polar body (**G**) and 4′,6-diamidino-2-phenylindole (DAPI) staining (**H**) of mature and immature oocytes. Scale bar = 2.0 mm. PB: Polar body; MII: metaphase II; GV: germinal vesicle. Superscripts with different letters indicate statistical significance.

To evaluate the prospective role of NAM during the process of oocyte maturation, the first dose–response experiment was performed using three low NAM concentrations (0.01, 0.1, and 1.0 mM) in addition to an untreated control. Microscopic investigation of COCs at the start and end of maturation showed a significant improvement in the expansion pattern of cumulus cells in 0.1 NAM-treated groups (30.20 ± 2.53 vs. 19.43 ± 2.19 for control) (Figure 1A,C). To investigate if increasing the concentration of NAM can further boost the expansion, another experiment was conducted using the three concentrations 10, 20, and 40 mM. Oppositely, high NAM concentrations, particularly 20 and 40 mM, severely interfered with the expansion in a dose-dependent manner (1.63 ± 0.34 and 0.82 ± 0.15, respectively; Figure 1B,D).

Additionally, the effect of low and high NAM concentrations on the IVM was firstly assessed through the direct investigation of polar body extrusion, 18–20 h from the onset of maturation, under microscope. As shown in Figure 1E, the percentages of oocytes with obvious polar body were high under the low NAM concentrations (76.00 ± 5.43, 86.66 ± 3.65, and 74.66 ± 5.75 for 0.01, 0.1, and 1.0 mM, respectively, vs. 69.30 ± 3.40 for control). Contrarily, the high doses of NAM significantly diminished the maturation of oocytes (57.38 ± 3.99, 44.02 ± 4.54, 34.66 ± 2.49, and 70.64 ± 2.67 for 10, 20, and 40 mM, and control, respectively; Figure 1F). For confirmation, the percentage of metaphase II (MII)-oocytes was investigated following DAPI staining. As seen in Figure 1E, NAM at 0.1 mM increased the percentage of oocytes that reached MII stage (80.00 ± 4.08 vs. 65.00 ± 6.45 for control; *p* > 0.05), whereas high NAM concentrations induced oocyte arrest at germinal vesicle (GV) stage (immature oocytes) and, hence, reduced the level of MII oocyte (52.50 ± 2.50, 40.00 ± 4.08, 30.00 ± 4.08m and 67.50 ± 4.79 for 10, 20, and 40 mM, and the control respectively; Figure 1F). Representative images for polar body extrusion, as well as MII and GV oocytes, are shown in Figure 1G,H.

**Figure 2:**
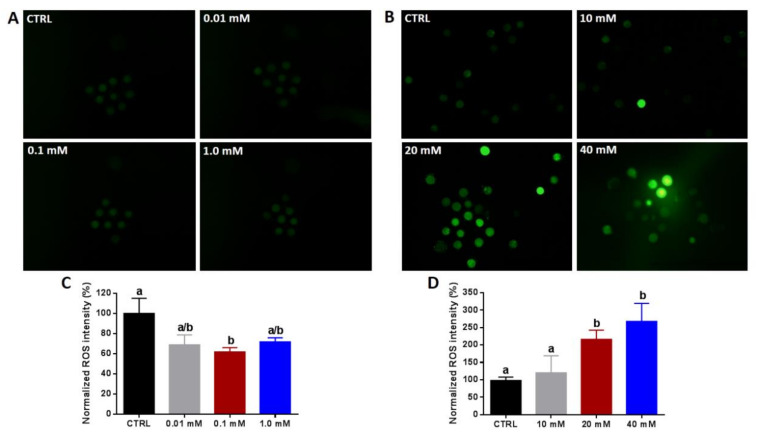
Determination of intracellular reactive oxygen species (ROS) under NAM treatment. 2,7-Dichlorodihydrofluorescein diacetate (H_2_DCFDA) staining of oocytes matured under low (**A**) and high (**B**) NAM concentrations. (**C**,**D**) The fluorescence intensity of ROS per individual oocyte analyzed by ImageJ. Original magnification 40×. Superscripts with different letters indicate statistical significance.

Next, the impact of low and high NAM concentrations on intracellular ROS signals, an indicator for oxidative stress, was explored at the end of the IVM using H_2_DCFDA staining. Exposure of oocytes to low NAM concentrations was accompanied by a decline in the ROS signals in all treated groups that reached significance under 0.1 mM treatment (Figure 2A,C). On the contrary, NAM at 20 and 40 mM significantly increased the ROS levels in a dose-dependent manner (Figure 2B,D).

### 3.2. Impact of NAM Administratration during the IVM on the Development and Hatching of Embryos

**Figure 3:**
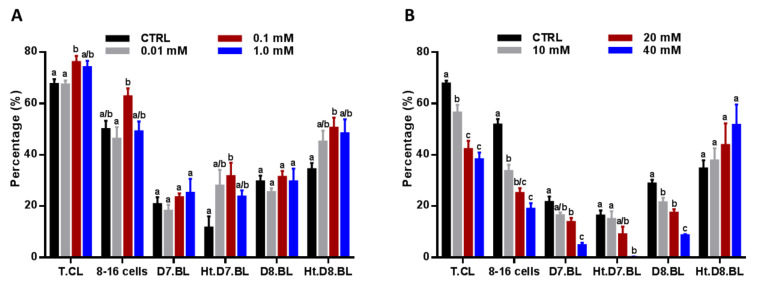
Effect of NAM administration during in vitro maturation (IVM) on the developmental competence of embryos. Day-4 total cleavage and 8–16 cell-stage embryos, and day-7 and day-8 blastocyst development and hatching rates were recorded in embryos derived from oocytes treated with low (**A**) and high (**B**) NAM concentrations. T.CL: day-4 total cleavage; D.7BL: day-7 blastocyst; Ht: hatched/hatching; D8.BL: day-8 blastocyst. Superscripts with different letters indicate statistical significance.

To investigate the effect of low and high NAM concentrations on the competence of embryo development, the total cleavage, 8–16 cell-stage embryos, and the early/late blastocyst development and hatching rates were recorded. Interestingly, administration of low NAM concentrations potentiated the day-4 total cleavage and 8–16 cell-stage embryo development rates that reached statistical significance, in the case of total cleavage, under 0.1 mM treatment: 76.17% ± 2.39% vs. 67.67% ± 1.84% for control for cleavage rate and 62.86% ± 3.04% vs. 50.00% ± 3.29% for control for 8–16 cell stage (Figure 3A). Although, checking the early and late blastocyst development rates at day 7 and day 8, respectively, did not show significant differences between groups, the hatching rates at the same time points were significantly improved under 0.1 mM NAM treatment (31.71% ± 5.15% vs. 11.71% ± 4.32% for control at day 7 and 50.57% ± 3.92% vs. 34.43% ± 2.39% for control at day 8; Figure 3A).

In contrast to low NAM concentrations, high NAM exposure settings significantly reduced the day-4 total cleavage and 8–16 cell-stage embryos in all treated groups in a dose-dependent manner (67.83% ± 1.11%, 56.50% ± 3.02%, 42.33% ± 3.18%, and 38.33% ± 2.59% for total cleavage and 51.83% ± 2.14%, 33.67% ± 2.50%, 25.17% ± 1.89%, and 19.00% ± 2.18% for 8–16 cell stage for 0.0, 10, 20, and 40 mM, respectively; Figure 3B). Additionally, the treatment significantly decreased the development of both day-7 (21.67% ± 2.08%, 16.50% ± 1.06%, 13.83% ± 1.58%, and 4.83% ± 0.95% for 0.0, 10, 20, and 40 mM, respectively) and day-8 (28.83% ± 1.47%, 21.50% ± 1.82%, 17.50% ± 1.36%, and 8.67% ± 0.42% for 0.0, 10, 20, and 40 mM, respectively) blastocysts (Figure 3B). Although the hatching rates at day 8 were slightly improved in the treated groups, this effect was non-significant (Figure 3B).

### 3.3. NAM Modulates Mitochondrial Content and Distribution Pattern in Oocytes

**Figure 4:**
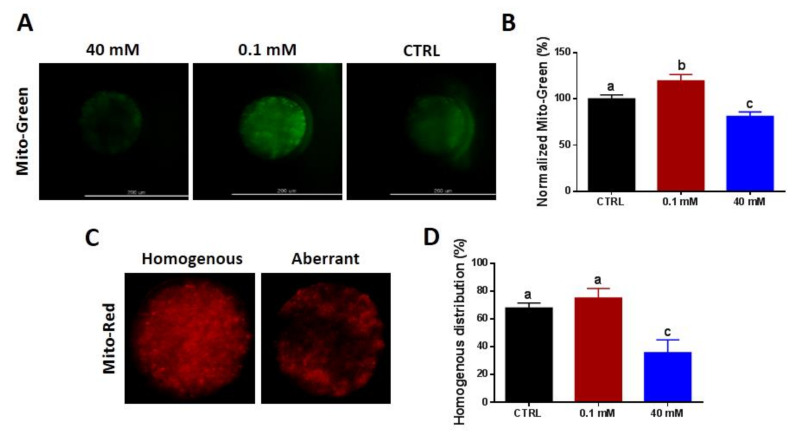
Mitochondrial content and distribution pattern following NAM treatment. Mito Tracker green staining (**A**) and integrated optical density (**B**) of oocyte mitochondrial content. (**C**) Mito Tracker Red staining for mitochondria showing the homogeneous and aberrant distribution patterns. (**D**) Proportion of oocytes with homogeneous mitochondrial distribution. Original magnification 200×. Mito-Green: Mito Tracker Green; Mito-Red: Mito Tracker Red. Superscripts with different letters indicate statistical significance.

Based on the aforementioned results, further experiments were performed using 40 and 0.1 mM, corresponding to high and low NAM concentrations, respectively, in addition to an untreated control. These two NAM concentrations were selected based on their paradoxical effects on cumulus expansion, oocyte maturation, ROS levels, and developmental competence of embryos. Using Mito Tracker Green staining, the integrated fluorescence intensity was significantly reduced in oocytes treated with 40 mM NAM, while it was significantly increased following 0.1 mM treatment (Figure 4A,B). Additionally, the majority of oocytes matured under 40 mM NAM treatment exhibited an aberrant distribution pattern and only 35.75% ± 9.22% showed a homogeneous distribution compared to 67.86% ± 3.57% of control (*p* < 0.05; Figure 4C,D). Despite the observed increase in the percentage of oocytes with homogeneously distributed mitochondria, under 0.1 mM NAM treatment (75.0% ± 6.84%), this was statistically non-significant.

### 3.4. Regulation of Apoptosis and Autophagy in NAM-Treated Oocytes

**Figure 5:**
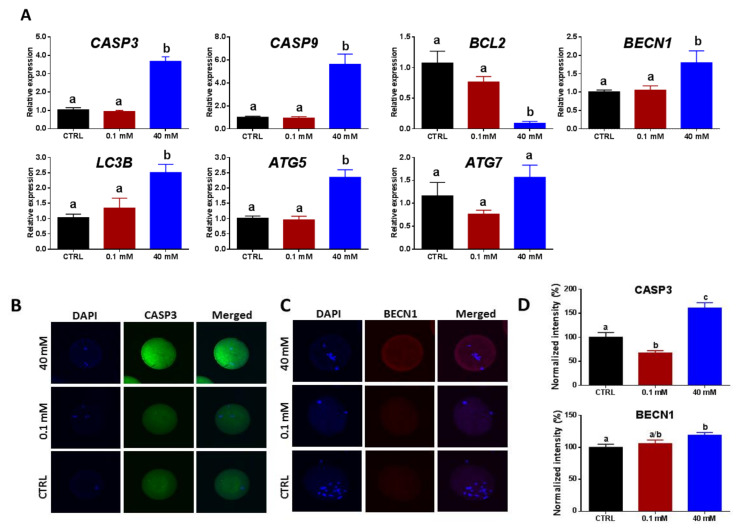
Effect of NAM addition during the maturation of oocytes on the levels of apoptosis and autophagy. (**A**) Relative expression of mRNA of different apoptosis- and autophagy-related genes. Immunofluorescence of CASP3 (**B**) and BECN1 (**C**) in oocytes treated with 0.1 and 40 mM NAM. (**D**) Integrated fluorescence intensities of CASP3 and BECN1 in oocytes. Original magnification 100×. Superscripts with different letters indicate statistical significance.

For assessment of the potential regulatory role of NAM on apoptosis and autophagy in oocytes, the expression levels of their specific markers were investigated at messenger RNA (mRNA) and/or protein levels. The results of RT-qPCR showed significant upregulation of the apoptosis-related genes *CASP3* and *CASP9* and downregulation of *BCL2* in 40 mM NAM-treated oocytes (Figure 5A). However, the mRNA levels of these genes did not show any significant difference between 0.1 mM NAM-treated and the control groups (Figure 5A). Furthermore, immunofluorescence of CASP3 showed overexpression in oocytes treated with 40 mM NAM and down-expression under 0.1 mM treatment (*p* < 0.05; Figure 5B,D). On the other hand, the mRNA levels of all autophagy-related markers (*BECN1*, *LC3B*, and *ATG5*), except *ATG7*, were significantly upregulated in 40 mM-treated oocytes (Figure 5A). In addition, testing the protein level of BECN1 showed significant overexpression under 40 mM NAM treatment, while the 0.1 mM group did not show any difference compared to the untreated control (Figure 5C,D).

### 3.5. Supplementation with Low and High NAM Concentrations during the IVM Paradoxically Affects DNA Fragmentation, Apoptosis, Autophagy, and the Quality of Developed Embryos

**Figure 6:**
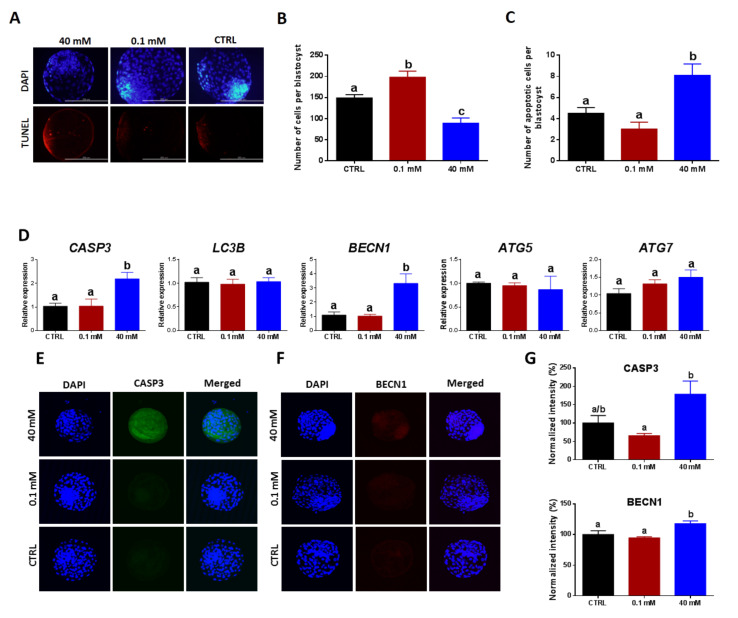
Effect of NAM administration during IVM on apoptosis- and autophagy-related genes/proteins in day-8 embryos. (**A**) Terminal deoxynucleotidyl transferase dUTP nick-end labeling (TUNEL) staining as indicator for DNA fragmentation in blastocysts; original magnification 400×. (**B**) The total number of cells and the number of apoptotic cells (**C**) per blastocyst. (**D**) Relative mRNA levels of different apoptosis (*CASP3*)- and autophagy (*LC3B, BECN1, ATG5,* and *ATG7*)-related genes. (**E**,**F**) Immunofluorescence of CASP3 and BECN1. (**G**) Integrated optical density of CASP3 and BECN1 in NAM-treated (40 and 0.1 mM) and control groups. Original magnification 100×. Superscripts with different letters indicate statistical significance.

To test if NAM exposure during the IVM of oocytes can affect the quality of preimplantation embryos, the total number of cells per blastocyst and the levels of apoptosis, as well as autophagy, were investigated. Using DAPI-based nuclei staining, the total number of cells per blastocyst was significantly higher in 0.1 mM NAM-treated group (197.7 ± 14.78) than the 40 mM-treated (89.17 ± 12.42) and the untreated control (148.8 ± 8.31) (Figure 6A,B). Additionally, the results of TUNEL assay, used for assessment of DNA fragmentation, showed a significant increase in the number of apoptotic cells in day-8 blastocysts derived from oocytes treated with 40 mM NAM compared to the control (8.10 ± 1.08 vs. 4.50 ± 0.54 for treated and control, respectively; Figure 6A,B), whereas blastocysts of 0.1 mM NAM treatment showed a lower incidence of DNA fragmentation (3.00 ± 0.67; Figure 6A,C).

Using RT-qPCR, the mRNA levels of *CASP3* in day-8 blastocysts were significantly upregulated following 40 mM NAM treatment, whereas the low NAM concentration (0.1 mM) did not show any significant difference, compared to control (Figure 6D). For corroboration, testing the protein level of CASP3 using immunofluorescence showed a significant overexpression in the 40 mM NAM-treated group (Figure 6E,G). Despite the observed decrease in CASP3 level in the 0.1 mM NAM-treated group, this effect did not reach statistical significance. On the other hand, the levels of autophagy-related markers *BECN1*, *LC3B*, *ATG5*, and *ATG7* were also inspected at transcriptional and/or translational levels. As shown in Figure 6D, only *BECN1* was significantly upregulated in the 40 mM-treated group, while the 0.1 mM NAM treatment did not affect the expression level of the tested genes. Additionally, the 40 mM NAM-derived embryos displayed a higher protein level of BECN1 compared to the slight decrease observed in 0.1 mM NAM-treated group (Figure 6F,G).

### 3.6. NAM Induces Hatching of Embryos, Derived from NAM-Treated Oocytes, Potentially via the Regulation of SIRT1 and PI3K/AKT/mTOR Signaling

**Figure 7:**
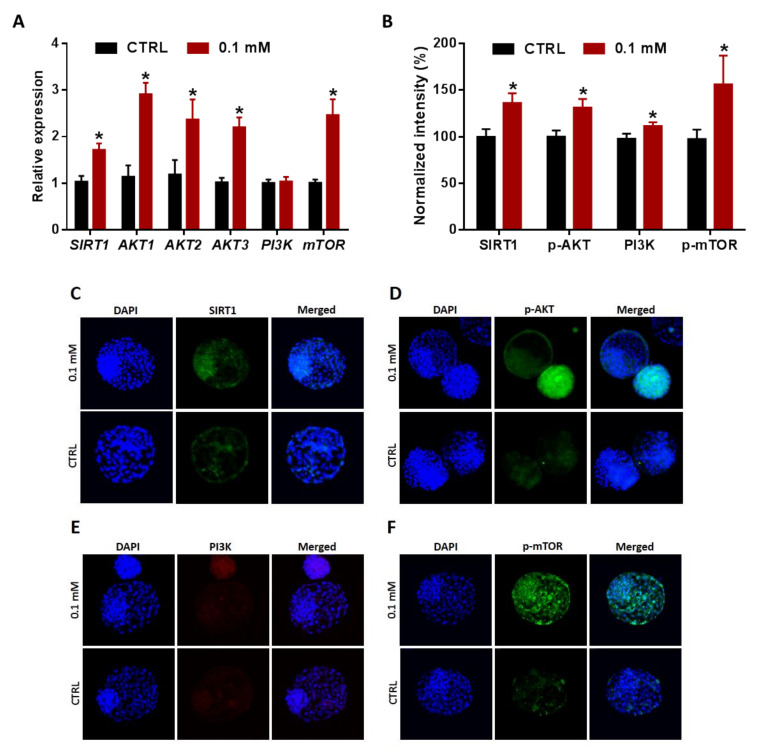
Expression of SIRT1, AKT, PI3K, and mTOR in day-8 blastocysts. (**A**) The mRNA levels of *SIRT1, AKT1, AKT2, AKT3, PI3K*, and *mTOR* in embryos developed from oocytes treated with 0.1 mM NAM. (**B**) Integrated optical density of SIRT1, p-AKT-Ser473, PI3K, and p-mTOR in NAM-treated and control embryos. Immunofluorescence of SIRT1 (**C**), p-AKT-Ser473 (**D**), PI3K (**E**), and p-mTOR (**F**) in blastocysts. Original magnification 100×. The asterisk indicates statistical significance.

Based on the above-mentioned data, the 0.1 mM NAM concentration was apparently recognized as the optimum concentration ensuring competent embryonic development and hatching in particular. We next sought to underline the possible mechanisms behind NAM-improved hatching using the 0.1 mM NAM concentration head to head with the untreated control. Previously, it was reported that SIRT1 and AKT can regulate the development and hatching of embryos. Our RT-qPCR and immunofluorescence results showed significant upregulation in the mRNA of *SIRT1, AKT1, AKT2*, and *AKT3* (Figure 7A), as well as the protein levels of SIRT1 and phosphorylated AKT (p-AKT-Ser473), in the NAM-treated group compared to control (Figure 7B–D). Likewise, PI3K and mTOR (effectors up- and downstream the AKT), considered as essential indicators for the survival and differentiation of embryos, were investigated in day-8 blastocyst at both mRNA and protein levels. As seen in Figure 7A, significant upregulation in the mRNA of *mTOR* was observed in embryos developed from NAM-treated oocytes compared to the untreated control, whereas comparable levels of *PI3K* were observed in the treated and untreated groups. However, the protein levels of both proteins were significantly higher in the NAM group compared to control (Figure 7B,E,F).

### 3.7. Lineages Specification of Embryos Developed from NAM-Treated Oocytes

**Figure 8:**
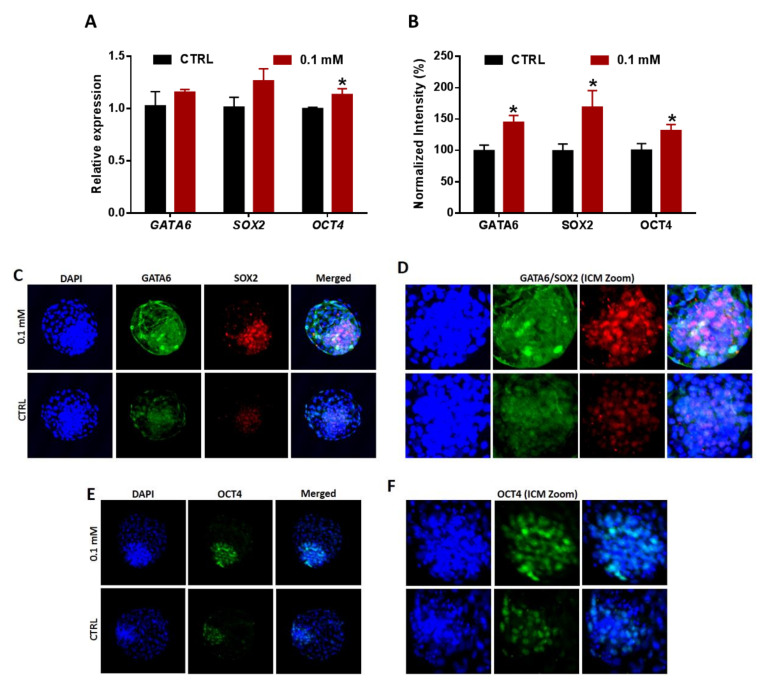
Transcriptional and translational levels of various factors related to ICM pluripotency in day-8 embryos. (**A**) The mRNA levels of *GATA6*, *SOX2*, and *OCT4* in treated and control embryos. (**B**) Integrated optical density of GATA6, SOX2, and OCT4. (**C**) Laser scanning confocal microscopy of GATA6 and SOX2. (**D**) GATA6 and SOX2 signal zoom showing localization in the ICM. (**E**) Immunofluorescence of OCT4 in day-8 blastocyst. (**F**) Zoom of OCT4 signal localized in the ICM cells. Original magnification 100×. The asterisk indicates statistical significance.

Finally, due to the critical role of PI3K/AKT/mTOR signaling in regulating the survival and proliferation, the levels of different lineage differentiation markers were delineated, following NAM treatment, using RT-qPCR and immunofluorescence. The mRNA of the PE-specific marker *GATA6* and the Epi-related markers *SOX2* and *OCT4*, having roles in the maintenance of ICM lineage segregation, were upregulated in NAM-treated groups, but a significant effect was observed only in *OCT4* (Figure 8A). However, the protein levels of the three markers were significantly higher in day-8 blastocysts of the NAM-treated group compared to the control (Figure 8B–F).

## 4. Discussion

The accumulation of ROS and the induction of oxidative stress, DNA fragmentation, and apoptosis are key hurdles for the production of high-quality embryos, a keystone for successful implantation in ART [2]. Despite the prevalent use of NAM as a therapeutic vitamin for a variety of health conditions, its role during embryo development is not completely clarified. The aim of the current study was to elucidate the effect of NAM supplementation, during IVM, on the developmental competence of bovine preimplantation embryos. A wide range of NAM concentrations was initially used, and the processes of cumulus expansion, oocyte maturation, oxidative stress, and embryo development were recorded. At the end of maturation, high concentrations of NAM (20 and 40 mM) negatively affected the processes of expansion and maturation, while the low concentration 0.1 mM improved them. Previously, the use of 5 and 10 mM NAM impaired the maturation of mouse and porcine oocytes, whereas the beneficial and non-effective roles of NAM were also reported [10,11,37,38], which support our finding. In line with these results, exposure of COCs to high NAM concentrations (above 10 mM) increased the level of ROS that was significantly reduced under 0.1 mM treatment. Similarly, the administration of high doses of NAM post-fertilization induced the accumulation of ROS in 2 cell-stage embryos [7]. Nonetheless, low ROS levels in T cells following treatment with 5 mM NAM were also reported [5,6], reflecting the antioxidant activity of low NAM concentrations.

Since oxidative stress and apoptosis in oocytes can subsequently influence embryonic development, fetal growth, and the health status of offspring [23,39,40], we sought to study the effect of NAM on the developmental competence of bovine embryos. Checking day-4 total cleavage and 8–16 cell stage embryos, as well as day-7 and day-8 blastocyst development rates, showed the detrimental effects of high NAM concentrations. These results are supported by the study of Yuan et al. showing the anti-developmental effect of 20 and 40 mM NAM when administered post-fertilization [7]. However, the use of NAM at low concentrations, 0.1 mM in particular, significantly improved the total cleavage and the hatching rate of embryos. We went further to clarify the distinction between high and low NAM exposure consequences. Previously, the relationship between NAM and the function of mitochondria was addressed in non-embryonic cells [5,6]. Due to the importance of mitochondria for energy storage and the normal physiology of oocytes, an improvement in mitochondrial profile under 0.1 mM NAM treatment was observed compared to the negative effect of 40 mM NAM reinforcing the data of expansion, maturation, and embryo development. Likewise, the interplay between NAM and autophagy was reported in different cells, but not yet clarified in the context of embryo development [5,6]. Exposure of oocytes to toxic compounds such as paraquat and glyphosate significantly reduced oocyte maturation and embryo development through activation of autophagy [13,14]. Contrarily, treatment with rapamycin, an mTOR inhibitor, induced the autophagy and the development of embryos [16]. In our study, the autophagy markers *BECN1, LC3B, ATG5*, and *ATG7* were upregulated under 40 mM NAM, albeit not affected by 0.1 mM NAM treatment, confirming the detrimental effect of high NAM concentrations. Noteworthy, proteins belonging to the anti-apoptotic BCL2-protein family have the ability to suppress autophagy through binding with the autophagy-related protein BECN1 highlighting the crosstalk between autophagy and apoptosis [41,42]. In line with this, the 40 mM NAM treatment significantly downregulated the *BCL2* transcription and upregulated *CASP3* and *CASP9*, while the low NAM concentration significantly reduced the level of CASP3 in oocytes. Similarly, the apoptosis profile in the developed blastocysts showed overexpression of CASP3 under 40 mM and lower expression in the 0.1 mM treatment, endorsing the apoptosis-inducing potential of high NAM concentrations. This also matches with the results of oocytes and confirms the unfavorable over-activation of autophagy following the exposure to high NAM concentrations.

It was reported that the success of IVF therapy correlates with the production of high-quality embryos displaying normal developmental competence, normal morphology, and low incidence of apoptosis [43]. Furthermore, the quality of blastocysts can be significantly affected by the treatments during the process of IVM [44]. In this regard, the TUNEL assay is universally used to detect DNA fragmentation and apoptosis in cells and, hence, evaluate the quality of preimplantation embryos [15,30]. In the current study, blastocysts corresponding to the group of low NAM exposure setting exhibited normal morphology with a significant increase in the total number of cells and lower incidence of DNA fragmentation, whereas the opposite profile was observed in the high NAM concentration group, confirming the data on apoptosis and autophagy.

Due to the importance of hatching for successful implantation and pregnancy [28,29], we moved forward to delineate the possible mechanisms behind the improved hatching witnessed following NAM treatment. Previously, supplementation of IVM medium with the SIRT1 activator resveratrol improved the quality and the hatching of blastocysts [3]. On the other hand, Riley et al. reported that inhibition of AKT significantly affected the normal physiology and hatching of blastocysts [22], while silencing the function of fatty acid synthase, an essential enzyme catalyze biogenesis of fatty acids, negatively influenced the hatching of embryos mainly through the downregulation of AKT [30]. Importantly, the activation of AKT/mTOR signaling in mouse oocytes and primordial follicles following the administration of resveratrol, an SIRT1 activator, was reported [19]. However, endoplasmic reticulum stress-induced SIRT1 upregulation was mediated by PI3K/AKT/GSK3β signaling in non-embryonic cells [45]. To explore the possible linkage between NAM and SIRT1/AKT signaling during the hatching of embryos, the expression levels of these two proteins were investigated in day-8 blastocysts. Intriguingly, embryos developed from NAM-treated oocytes exhibited high levels of SIRT1 and AKT at both mRNA and protein levels which might explain the improved hatching. Although the observed activation of SIRT1 by NAM is supported by several studies [46,47,48,49], this finding is also contradicted by others that showed an inhibitory effect of NAM on SIRT1 [1]. This discrepancy could be attributed to several factors including differences in the used cells, the dose/duration of treatment, and the time of investigation post-NAM administration. Additionally, the conversion of NAM to NAD, through the salvage pathway which is mediated by the enzymatic activity of nicotinamide phosphoribosyltransferase (NAMPT), required for SIRT1 activity, could also be a factor, since the continuous exposure to NAM increased the concentration of NAD by around 40% that was maintained for 21 days [46,47,50,51]. Notably, in a study showing the upregulation of SIRT1 by NAM, the inhibition of NAMPT prevented the induction of NAD by NAM but failed to abolish the upregulation of SIRT1, suggesting a partial regulation of SIRT1 by NAD [46]. This necessitates more in-depth studies to dissect the multiple effects of NAM on different intracellular signaling pathways, considering the levels of SIRT1 and NAD.

**Figure 9:**
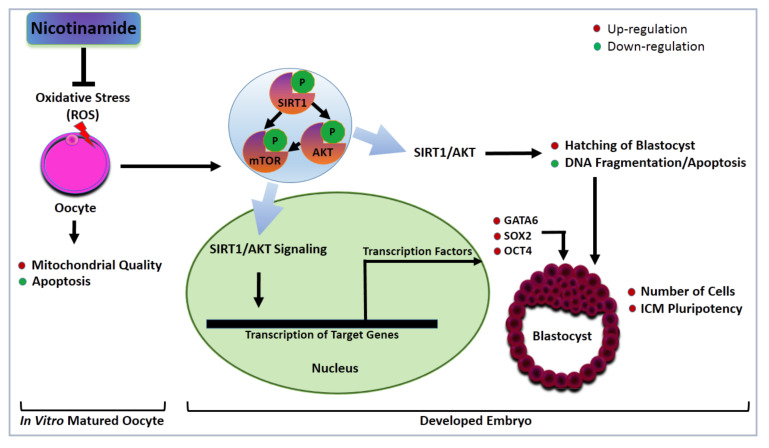
Proposed mechanism for the effect of NAM supplementation during IVM on the developmental competence of bovine embryos. Low NAM concentrations can reduce oxidative stress and apoptosis and improve mitochondrial function in oocytes. The high quality and hatching rate of embryos developed from NAM-treated oocytes could be accredited to the regulation of SIRT1/AKT signaling, as well as the transcription factors GATA6, SOX2, and OCT4.

The critical role of PI3K/AKT/mTOR signaling in controlling the survival and proliferation of certain cells such as neurons was previously described [52,53]. In our study, upregulation of PI3K/AKT/mTOR and the lower incidence of apoptosis were observed in embryos developed from low NAM-treated oocytes. This matches with the study of Chong et al. that showed an enhancement of neural cell survival following administration of NAM under anoxia conditions through activating the AKT signaling that consequently reduced apoptosis [53]. Moreover, PI3K/AKT/mTOR signaling has a significant role in the development, metabolism, and pluripotency fate of cells [22,24,25] via modulating different transcription factors such as OCT4, SOX2, GATA4 and GATA6 [34,35]. OCT4 is the master controller of lineage specification of ICM [54] and for embryonic lineage-specific markers including SOX2 and GATA6. Importantly, GATA6 expression was absent in the ICM of the OCT4-null embryos [34]. In addition, OCT4 and SOX2 transcriptional factors are necessary to maintain the pluripotent state of Epi ICM lineages [35], while GATA4 and GATA6 are key determinants of the PE fate [34]. Intriguingly, one common feature shared by OCT4 and SOX2 is that they have a consensus AKT phosphorylation motif which further consolidates the critical role of AKT signaling as a pluripotency factor during the development of preimplantation embryos [55,56]. Similar to these studies, we found significant upregulation of OCT4, SOX2, and GATA6 in the ICM of NAM-treated group highlighting the involvement of NAM in the maintenance of ICM lineage specifications. The proposed mechanisms of NAM’s role during blastocyst hatching and differentiation maintenance are summarized in Figure 9.

## 5. Conclusions

Collectively, the exposure of bovine oocytes to low concentrations of NAM can improve the developmental competence of preimplantation embryos through reducing the oxidative stress, DNA fragmentation, and apoptosis. Furthermore, to the best of our knowledge, this is the first study reporting that NAM supplementation, during the process of IVM, can improve the hatching and differentiation of embryos, paving the way for more in-depth studies on deciphering the multiple molecular mechanisms behind the hatching process that could be useful in assisted reproductive technology.

## Figures and Tables

**Table 1:** Sequences and amplification sizes of the primers used in RT-qPCR analysis.

Gene Name	Sequence	GenBank Accession Number	Product Size (bp)
Apoptosis/Autophagy-Related Genes			
*CASP3*	F: CCCAAGTGTGACCACTGAAC R: CCATTAGGCCACACTCACTG	NM_001077840.1	169
*CASP9*	F: CGCCACCATCTTCTCCCTG R: CCAACGTCTCCTTCTCCTCC	NM_001077111.1	83
*BCL2*	F: TGGATGACCGAGTACCTGAA R: CAGCCAGGAGAAATCAAACA	NM_001166486.1	120
*ATG5*	F: CCACTGCCGTCATTAAACCT R: TTCCACTCCCTCGAGCTAAA	XM_024996700.1	212
*ATG7*	F: ATGGCCTTTGAGGAACCTTT R: ATGCCTCCCTTCTGGTTCTT	XM_010817935.3	210
*BECN1*	F: AGTTGAGAAAGGCGAGACAC R: GATGGAATAGGAACCACCAC	NM_001033627.2	100
*LC3B*	F: TTATCCGAGAGCAGCATCC R: AGGCTTGATTAGCATTGAGC	NM_001001169.1	171
**SIRT1/AKT Signaling-Related Genes**			
*SIRT1*	F: CAACGGTTTCCATTCGTGTG R: GTTCGAGGATCTGTGCCAAT	NM_001192980.3	138
*PI3K*	F: TCAACCATGACTGTGTGCCA R: CCATCAGCATCAAATTGGGCA	XM_027540417.1	234
*AKT1*	F: AAAAGGAAGTGGTGTACAGG R: GAAGTCGGTGATCTTGATGT	NM_173986.2	80
*AKT2*	F: CGACTATCTCAAACTCCTGG R: ATCTTCATGGCATAGTAGCG	NM_001206146.2	90
*AKT3*	F: AGCTGTTTTTCCATTTGTCG R: TGTAGATAGTCCAAGGCAGA	NM_001191309.1	94
*mTOR*	F: TTAACAGGGTTCGAGAGAAG R: AGAGGTTTTCATGGGATGTC	XM_002694043.6	113
**ICM Pluripotency-Related Genes**			
*GATA6*	F: AAGATGCTGACCAGACATCT R: AGAGACCAGCTGCCTGGAAGT	XM_002697727.3	206
*SOX2*	F: CGAGTGGAAACTTTTGTCCG R: GGTATTTATAATCCGGGTGTT	NM_001105463.2	101
*OCT4*	F: GGAGAGCATGTTCCTGCAGTGC R: ACACTCGGACCACGTCCTTCTC	NM_174580.3	95
**Reference Genes**			
*GADPH*	F: CCCAGAATATCATCCCTGCT R: CTGCTTCACCACCTTCTTGA	NM_001034034.2	185
*ACTB*	F: ATTTTGAATGGACAGCCATC R: TGTACAGGAAAGCCCTGACT	NM_173979.3	120

## Supplementary Materials

The following are available online at https://www.mdpi.com/2073-4409/9/6/1550/s1: Table S1: Antibodies used in the study.
