# Supplementary material for: Nicotinamide Supplementation during the In Vitro Maturation of Oocytes Improves the Developmental Competence of Preimplantation Embryos: Potential Link to SIRT1/AKT Signaling
Source: Cells. 2020 Jun 25;9(6):1550. doi: 10.3390/cells9061550 (PMC7348965; doi:10.3390/cells9061550)
Supplement: Supplementary file 1 [file cells-09-01550-s001.pdf]

Table S1: Antibodies used in the study

	Manufacturer	Catalog number	Dilution
**Primary Antibodies**			
Anti-Caspase-3	Santa Cruz	Sc-1225	1:100
Anti-Beclin-1	Santa Cruz	Sc-48341	1:200
Anti-Sirt1	Cell Signaling	9475	1:200
Anti-PI3K	Santa Cruz	Sc-374534	1:100
Anti-p-Akt (Ser473)	Cell Signaling	4060	1:200
Anti-p-mTOR (S2448)	Abcam	ab84400	1:200
Anti-GATA6	Invitrogen	PA5-40438	1:250
Anti-SOX2	Santa Cruz	Sc-365964	1:200
Anti-OCT4	Invitrogen	PA5-27438	1:200
**Secondary Antibodies**			
(TRITC)-conjugated goat anti-rabbit IgG	Sigma-Aldrich	T6778	1:400
(FITC)-conjugated goat anti-mouse IgG	Santa Cruz	Sc-2010	1:200
(TRITC)-conjugated goat anti-mouse IgG	Invitrogen	A16071	1:250
Alexa Fluor-568 donkey anti-mouse IgG	Invitrogen	A10037	1:500
Alexa Fluor-488 donkey anti-rabbit IgG	Thermo Fisher	A21206	1:500
Alexa Fluor-488 donkey anti-goat IgG	Invitrogen	A11055	1:500
